# Outcomes and Prognostic Factors for Locally Recurrent Rectal Cancer Treated With Proton Beam Therapy

**DOI:** 10.1016/j.adro.2023.101192

**Published:** 2023-02-06

**Authors:** Yoshiaki Takagawa, Motohisa Suzuki, Hisashi Yamaguchi, Ichiro Seto, Yusuke Azami, Masanori Machida, Kanako Takayama, Takuya Tominaga, Masao Murakami

**Affiliations:** a Department of Minimally Invasive Surgical and Medical Oncology, Fukushima Medical University, Fukushima, Japan; b Department of Radiation Oncology, Southern TOHOKU Proton Therapy Center, Fukushima, Japan

## Abstract

**Purpose:**

Our objective was to report the outcome and prognostic factors for patients with locally recurrent rectal cancer (LRRC) treated with proton beam therapy (PBT) at our institution.

**Methods and Materials:**

The study included PBT-treated patients with LRRC between December 2008 and December 2019. Treatment response was stratified using an initial imaging test after PBT. Overall survival (OS), progression-free survival (PFS), and local control (LC) were estimated using the Kaplan-Meier method. Each outcome's prognostic factors were verified using the Cox proportional hazards model.

**Results:**

Twenty-three patients were enrolled (median follow-up, 37.4 months). There were 11 patients with complete response (CR) or complete metabolic response (CMR), 8 with partial response or partial metabolic response, 2 with stable disease or stable metabolic response, and 2 with progressive disease or progressive metabolic disease. Three- and 5-year OS, PFS, and LC were 72.1% and 44.6%, 37.9% and 37.9%, and 55.0% and 47.2%, respectively, with 54.4 months’ median survival time. The maximum standardized uptake value of fluorine-18-fluorodeoxyglucose-positron emission tomography–computed tomography (^18^F-FDG-PET/CT) before PBT (cutoff value, 10) showed significant differences in OS (*P* = .03), PFS (*P* = .027), and LC (*P* = .012). The patients who achieved CR or CMR after PBT had significantly better LC than those with non-CR or non-CMR (hazard ratio, 4.49; 95% confidence interval, 1.14-17.63; *P* = .021). Older patients (aged ≥65 years) had significantly higher LC and PFS rates. Patients with pain before PBT and larger tumors (≥30 mm) also had significantly lower PFS. Of 23 patients, 12 (52%) experienced further local recurrence after PBT. One patient developed grade 2 acute radiation dermatitis. Regarding late toxicity, grade 4 late gastrointestinal toxic effects were recorded in 3 patients, in 2 of whom reirradiation was associated with further local recurrence after PBT.

**Conclusions:**

The results showed that PBT may have potential to be a good treatment option for LRRC. ^18^F-FDG-PET/CT before and after PBT may be useful for assessing tumor response and predicting outcomes.

## Introduction

Rectal cancer is a common type of malignancy, with approximately 730,000 new cases and 340,000 deaths worldwide by 2020.^1^ The standard of care for locally advanced rectal cancer is termed as total neoadjuvant therapy (TNT), which is neoadjuvant chemoradiation therapy (nCRT), followed by total mesorectal excision.^2^, ^3^, ^4^ Recently, a prospective, randomized phase 2 trial of TNT for rectal adenocarcinoma demonstrated that TNT achieved a high local control rate (94%).^5^ Moreover, nCRT followed by watch-and-wait also achieved organ preservation in half of the patients.^5^ However, despite optimal nCRT followed by total mesorectal excision, local recurrence rates remain between 5% and 18%.^5^, ^6^, ^7^, ^8^ For patients with locally recurrent rectal cancer (LRRC) treated with salvage surgery, complete surgical resection (R0 resection) has a survival advantage over R1 and R2 resection.^9^, ^10^, ^11^ However, curative surgery for LRRC, such as total pelvic exenteration, significantly impairs the patient's quality of life, and some patients prefer nonsurgical treatment for LRRC. Proton beam therapy (PBT) has unique physical characteristics, such as the Bragg peak. Therefore, PBT enables a higher dose for LRRC without severe toxic effects compared with conventional photon radiation therapy. This study retrospectively analyzed PBT outcomes for LRRC at our institution.

## Methods and Materials

### Patient selection

We included PBT-treated patients with LRRC between December 2008 and December 2019. We excluded patients with a history of salvage surgery, radiation therapy, or distant metastasis before PBT and those treated with postoperative adjuvant PBT. Patients with a history of chemotherapy were included. To determine the indications for PBT, fluorine-18-fluorodeoxyglucose-positron emission tomography–computed tomography (^18^F-FDG-PET/CT) was performed before PBT planning. This study was approved by the institutional review board.

### Proton beam therapy

Computed tomography planning was done at 2.0-mm intervals for PBT planning. When contouring of the recurrent tumor was unclear on CT, pelvic magnetic resonance imaging (MRI) was performed. The PBT machine used was the Hitachi proton‐type particle therapy system (Hitachi, Kashiwa, Japan). The PBT planning system used was the XiO‐M (Hitachi). Gross tumor volume (GTV) was defined as a recurrent tumor. The median clinical target volume (CTV) margin was 5 mm (range, 2-7 mm) around the GTV. The median planning target volume (PTV) margin was 5 mm (range, 3-10 mm) around the CTV. The supine position is the standard treatment position because of the high morbidity rate of the postoperative colonic stoma. Irradiation was performed 5 days a week and at least 4 days a week, even on holidays. PBT was administered using the passive scattering method. The basic policy of dose and fractionation at our institution was as follows: if possible, the dose per fraction was >2.2 Gy and the total dose was >70 Gy. The maximum doses for the small and large bowels were 50 and 60 Gy, respectively. Moreover, the dose for the bladder, urethra, pelvic bone, and skin was not over the 100% isodose line. However, the radiation oncologist in charge decided the radiation dose and fractionation based on tumor location, the distance between tumors and organs at risk, and the patient's condition. The relative biological effectiveness (RBE) value of 1.1 was used in this study. Replanning, including a boost plan to reduce dose to organs at risk, was adopted for most patients during PBT.

### Clinical response assessment

Treatment response was stratified using an initial imaging test after PBT. Imaging modality was used by CT or MRI or ^18^F-FDG-PET/CT. The response criteria in CT and MRI used response evaluation criteria in solid tumors (RECIST). The response criteria in ^18^F-FDG-PET/CT used the European Organisation for Research and Treatment of Cancer criteria.^12^ Complete resolution of FDG uptake in all lesions was determined as a complete metabolic response (CMR). A reduction of greater than 25% in the sum of the maximum standardized uptake value (SUV_max_) after PBT was determined as partial metabolic response (PMR). An increase of more than 25% in the sum of the SUV_max_ or the appearance of new FDG-avid lesions were defined as progressive metabolic disease (PMD). Not qualifying as CMR, PMR, or PMD was defined as stable metabolic disease.

### Statistical analysis

We estimated overall survival (OS), progression-free survival (PFS), and local control (LC) using the Kaplan-Meier method. The follow-up period started on the date of PBT completion. The prognostic factors for each outcome were verified using the Cox proportional hazards model. All statistical analyses were performed using Stata, version 11.1 (StataCorp LLC, College Station, TX).

## Results

**Figure 1:**
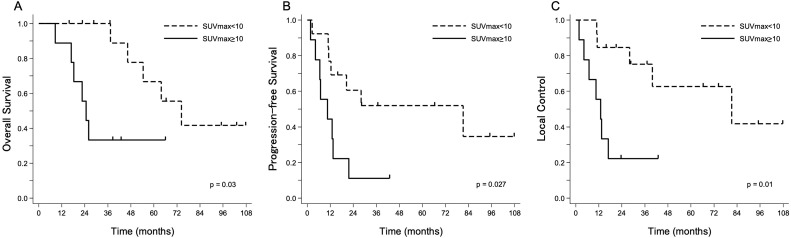
Comparison of (A) overall survival, (B) progression-free survival, and (C) local control between the group with low maximum standardized uptake value (SUV_max_ <10) before proton beam therapy (PBT) versus the high SUV_max_ group (SUV_max_ ≥10) (n = 22).

**Figure 2:**
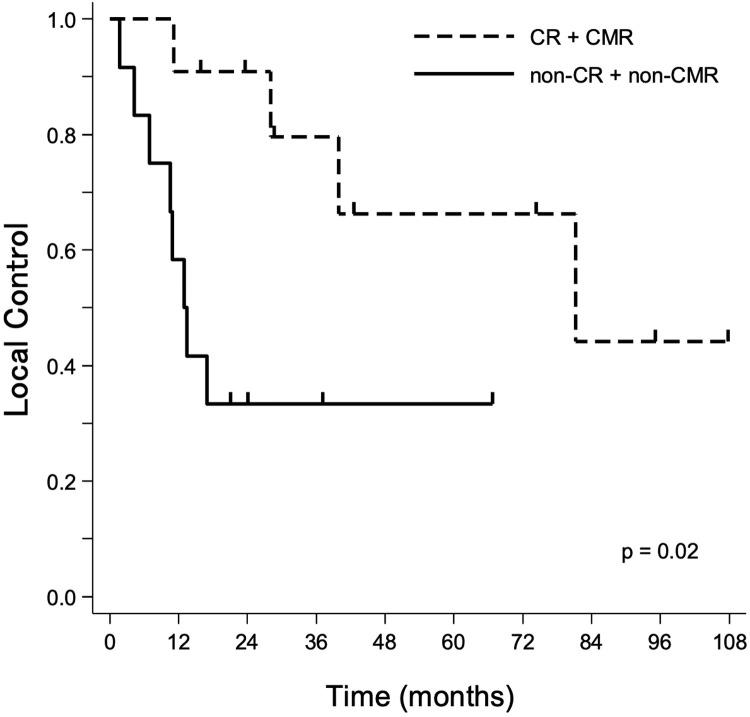
Kaplan-Meier curve of local control between the complete response (CR) or complete metabolic response (CMR) group by initial imaging response after proton beam therapy (PBT) versus the non-CR or non-CMR group (n = 23).

**Figure 3:**
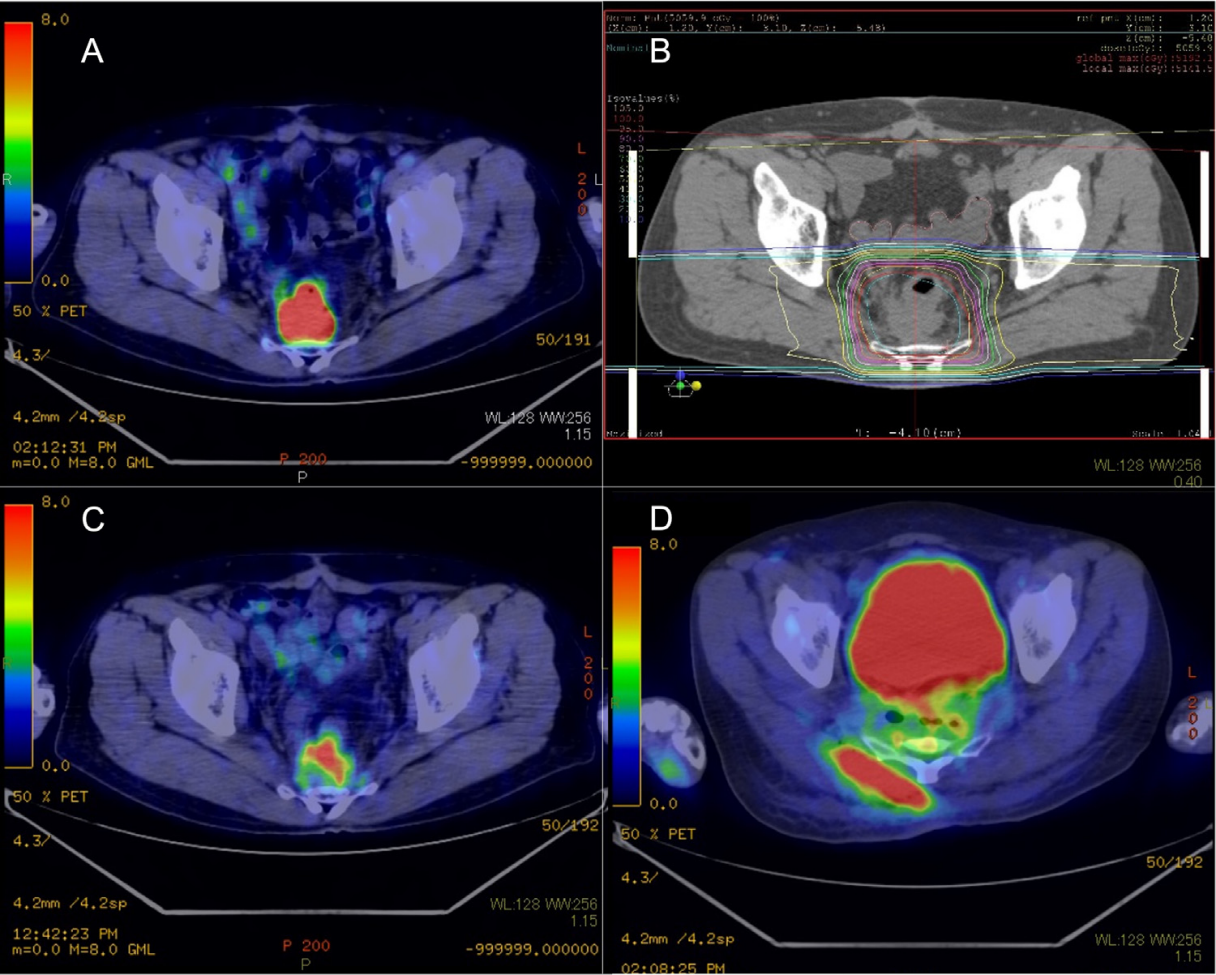
Representative case of a further local recurrence of locally recurrent rectal cancer treated with proton beam therapy (PBT). (A) Image of fluorine-18-fluorodeoxyglucose-positron emission tomography–computed tomography (^18^F-FDG-PET/CT) before PBT. The recurrent tumor showed a high maximum standardized uptake value (SUV_max_ = 22.5). (B) Image of dose distribution of PBT planning. (C) Image of ^18^F-FDG-PET/CT 2 months after PBT. The initial imaging response was a partial response. (D) Image of ^18^F-FDG-PET/CT 11 months after PBT. Further local recurrence occurred in the postsacral region, including the irradiated area.

Twenty-three patients were enrolled in this study. The median follow-up time was 37.4 months. The patient characteristics are summarized in Table 1. The median age of the patients was 64 years (range, 34-83 years). The median time from surgery to recurrence was 18.1 months (range, 6.7-255.8 months). Twenty patients (87%) had adenocarcinoma, 2 had carcinoid, and 1 had neuroendocrine carcinoma. Salvage surgery was not performed in 10 patients because they denied to undergo this surgery even though the recurrent tumor was operable, whereas the remaining 13 patients had an inoperable recurrent tumor. The postoperative recurrence sites were as follows: 12 patients had a recurrence in the presacral region, 7 patients in the pelvic sidewall, and 4 patients in anastomoses. The median maximum size of the recurrent tumor was 37 mm (range, 16-130 mm). Sixteen patients (69.5%) had a prior history of chemotherapy before PBT. To rule out distant metastases, 22 patients (95.6%) had received ^18^F-FDG-PET/CT before PBT. Only 1 patient did not receive ^18^F-FDG-PET/CT before PBT, owing to recurrence of the rectal carcinoid. The median SUV_max_ of ^18^F-FDG-PET/CT before PBT was 7.7 (range, 3.3-22.9). Twenty-one patients (91%) underwent PBT alone. One patient was administered concurrent chemotherapy (fluorouracil, calcium levofolinate hydrate, oxaliplatin, and bevacizumab), and the remaining patient received concurrent hyperthermia. Twenty-two patients were in the supine position as the treatment position, and 1 patient was in the prone position. The median total dose was 72.6 Gy (RBE) (range, 60-87 Gy). In this study, various dose-fractionation regimens were used. The dose-fractionation regimens are listed in Table 2. The initial imaging test was performed at a median of 2 months after PBT. Sixteen patients (70%) received ^18^F-FDG-PET/CT as the initial imaging modality. In 15 of the 16 patients, the median SUV_max_ of ^18^F-FDG-PET/CT after PBT was 3.9 (range, 1.9-15.0). Four patients underwent MRI, and the remaining 3 patients underwent CT as the initial imaging modality. Ten patients had CMR, 4 had PMR, and 2 had PMD. One patient had complete response (CR), 4 had partial response, and 2 had stable disease. Eleven patients (48%) experienced lumbar or anal pain before PBT and had symptomatic pain improvement after PBT. Three- and 5-year OS, PFS, and LC were 72.1% and 44.6%, 37.9% and 37.9%, and 55.0% and 47.2%, respectively. The median survival time was 54.4 months. Univariate analyses (UVA) using the Cox proportional hazards model for outcomes are summarized in Table 3. In the UVA, the SUV_max_ of ^18^F-FDG-PET/CT before PBT (cutoff value, 10) showed significant difference for OS (hazard ratio [HR], 4.14; 95% confidence interval [CI], 1.12-15.34; *P* = .03), PFS (HR, 3.37; 95% CI, 1.14-9.94; *P* = .027), and LC (HR, 4.91; 95% CI, 1.38-17.49; *P* = .012) (Fig. 1). The patients who achieved CR or CMR after PBT had significantly better LC compared with those with non-CR or non-CMR (HR, 4.49; 95% CI, 1.14-17.63; *P* = .021) (Fig. 2). Older patients (≥65 years of age) had significantly better LC (HR, 0.13; 95% CI, 0.03-0.62; *P* = .0024) and PFS (HR, 0.30; 95% CI, 0.10-0.89; *P* = .024). The patients with pain before PBT and larger tumors (≥30 mm) also had significantly lower PFS (HR, 3.20 [95% CI, 1.09-9.41]; *P* = .028; and HR, 3.35 [95% CI, 0.93-12.03]; *P* = .04, respectively). The total radiation dose (cutoff value: biologically effective dose [BED] was 90 Gy, α/β = 10), recurrent site (lateral vs central), and recurrent tumor size (cutoff value, 30 mm) did not show a significant difference in all outcomes. Multivariate analyses were not performed owing to the small sample size. Of 23 patients, 12 (52%) experienced further local recurrence after PBT. Representative cases of further local recurrence of LRRC treated with PBT are shown in Fig. 3. One patient developed grade 2 acute radiation dermatitis. Grade 4 late gastrointestinal toxic effects were recorded in 3 patients (Figs. E1-E3), in 2 of whom reirradiation was associated with further local recurrence after initial PBT. The remaining patient developed perforation of the ileum 8 months after receiving 72 Gy (RBE) in 30 fractions of PBT (Fig. E1). Partial resection of the ileum was performed, and the perforated ileum was close to the irradiation field. The patient had a history of bevacizumab use before PBT.

**Table 1:** Patient and treatment characteristics

Patient number	23
Age (y), median (range)	64 (34–83)
Sex	
Female	10
Male	13
ECOG performance status score	
0	21
1	2
Surgery	
APR	9
Hartmann	5
LAR	6
Transanal resection	2
Unknown	1
Histology	
Adenocarcinoma	20
Carcinoid	2
Neuroendocrine carcinoma	1
Primary tumor stage*	
I	2
II	6
III	8
Unknown	7
Postoperative recurrent site	
Presacral	12
Pelvic side wall	7
Anastomosis	4
Recurrent tumor size (mm), median (range)	37 (16–130)
SUV_max_ of ^18^F-FDG-PET/CT, median (range)	7.7 (3.3–22.9)
Treatment	
PBT alone	21
Concurrent chemotherapy	1
Concurrent hyperthermia	1
Total dose (Gy, RBE), median (range)	72.6 (60–87)

*Abbreviations:*^18^F-FDG-PET/CT = fluorine-18-fluorodeoxyglucose-positron emission tomography–computed tomography; APR = abdominal perineal resection; ECOG = Eastern Cooperative Oncology Group; LAR = low anterior resection; PBT = proton beam therapy; RBE = relative biological effectiveness; SUV_max_ = maximum standardized uptake value.

⁎ Union for International Cancer Control classification, seventh edition.

**Table 2:** Characteristics of dose fractionation regimens

Total dose, Gy, RBE	Fractionation	Number	BED_10_
60.0	30	1	72.0
61.6	28	1	75.1
62.0	31	1	74.4
68.2	31	1	83.2
70.0	35	2	84.0
70.4	32	1	85.9
72.0	30	3	89.3
72.6	33	3	88.5
74.8	34	2	91.2
75.0	25	3	97.5
75.0	30	2	93.7
77.0	35	2	93.9
87.0 (75.0 + 12.0)	33 (30 + 3)	1	110.5 (93.7 + 16.8)

*Abbreviations:* BED_10_ = biologically effective dose for an α/β ratio of 10; RBE = relative biological effectiveness.

**Table 3:** Prognostic factors for OS, PFS, and LC using univariate analysis

Factor	OS		PFS		LC	
	HR (95% CI)	*P* value	HR (95% CI)	*P* value	HR (95% CI)	*P* value
Age (≥65 y vs <65 y)	0.38 (0.11–1.33)	.12	0.30 (0.10–0.89)	.024	0.13 (0.03–0.62)	.0024
Sex (male vs female)	1.16 (0.32–4.16)	.82	1.47 (0.51–4.20)	.47	1.66 (0.50–5.53)	.42
Total radiation dose (≥BED_10_ 90 Gy vs < BED_10_ 90 Gy)	0.75 (0.21–2.65)	.65	0.70 (0.25–1.98)	.49	0.54 (0.16–1.82)	.31
Recurrent site (lateral vs central)	0.83 (0.17–3.99)	.81	0.59 (0.16–2.11)	.39	1.03 (0.27–3.92)	.96
Recurrent tumor size (≥30 mm vs <30 mm)	3.37 (0.72–15.79)	.085	3.35 (0.93–12.03)	.04	2.14 (0.57–8.08)	.23
SUV_max_ of ^18^F-FDG-PET/CT before PBT (≥10 vs <10)	4.14 (1.12–15.34)	.03	3.37 (1.14–9.94)	.027	4.91 (1.38–17.49)	.012
Pain before PBT (yes vs no)	2.50 (0.65–9.54)	.16	3.20 (1.09–9.41)	.028	2.39 (0.71–7.98)	.14
Time from surgery to recurrence (≥2 y vs <2 y)	0.57 (0.16–1.95)	.36	0.83 (0.30–2.31)	.72	0.79 (0.25–2.52)	.69
Chemotherapy before PBT (yes vs no)	0.63 (0.17–2.27)	.49	1.37 (0.42–4.41)	.59	1.44 (0.38–5.47)	.58
Initial imaging response after PBT (CR + CMR vs non-CR + non-CMR)	2.04 (0.57–7.32)	.27	2.47 (0.82–7.45)	.099	4.49 (1.14–17.63)	.021

*Abbreviations:*^18^F-FDG-PET/CT = fluorine-18-fluorodeoxyglucose-positron emission tomography–computed tomography; BED_10_ = biologically effective dose for an α/β ratio of 10; CI = confidence interval; CMR = complete metabolic response; CR = complete response; HR = hazard ratio; LC = local control; OS = overall survival; PBT = proton beam therapy; PFS = progression free survival; SUV_max_ = maximum standardized uptake value.

## Discussion

We demonstrated promising outcomes in PBT-treated patients with LRRC. Additionally, ^18^F-FDG-PET/CT before and after PBT may be useful for assessing tumor response and predicting outcomes. There are few studies to examine prognostic factors for the patients with LRRC treated with PBT.

Locally recurrent rectal cancer exhibits very low radiosensitivity. Tanaka et al reported that 3-year OS and LC rates with 3-dimensional conformal radiation therapy were 45.2% and 19.6%.^13^ A phase 2 study of concurrent capecitabine and irinotecan with intensity modulated radiation therapy for recurrent rectal cancer reported 3-year OS and local progression-free survival rates of 36.5% and 33.9%, respectively.^14^ Based on these reports, chemoradiation therapy (CRT) using the intensity modulated radiation therapy technique for LRRC had insufficient outcomes. In contrast, there are few reports of stereotactic body radiation therapy for LRRC. Kim et al reported good 4-year local control rates (74.3%) with a median follow-up time of 31 months for 23 patients with pelvic lymph node recurrence of rectal cancer using the stereotactic body radiation therapy technique with CyberKnife (Accuray Inc, Sunnyvale, CA).^15^ However, they excluded the patients whose tumors recurred in the anastomosis site and in the residual colon. In addition, their 4-year OS rates were low (24.9%). There are a small number of studies on PBT for LRRC. In 2012, Hamauchi et al reported PBT with 70 Gy (RBE) in 25 fractions for 13 patients with LRRC with a median follow-up time of 42 months, showing a 46% LC rate with less severe toxicity.^16^ Hiroshima et al reported that 12 patients with LRRC were treated with PBT.^17^ They showed the 3-year OS, PFS, and LC were 71.3%, 12.1%, and 80.2%, respectively. In their study, 6 of the 12 patients received concurrent S-1 chemotherapy during PBT. The outcomes of the current study were comparable to those of previous studies. To our knowledge, this is the largest study of patients with LRRC treated with PBT. However, in our study, approximately 50% of patients experienced further local recurrence after PBT. Concurrent chemotherapy with PBT may further improve the LC rate in patients with LRRC.

In contrast, carbon-ion radiation therapy (CIRT) has a higher BED than PBT. There have been 2 reports of CIRT for LRRC in Japan. Yamada et al reported the 3-year and 5-year OS rates were 88% and 59% using 73.6 Gy (RBE) in 16 fractions regimen.^18^ Shinoto et al reported a multi-institutional study of CIRT for LRRC.^19^ They demonstrated the 3-year and 5-year OS rates were 73% and 51%, respectively, in 224 patients with LRRC. Most of the patients (98%) were using a regimen of 73.6 Gy (RBE) in 16 fractions; the researchers documented that grade 3 acute toxic effects were observed in 3 patients (1%), and grade 3 late toxic effects were observed in 12 patients (5%). These studies on CIRT for LRRC demonstrated favorable outcomes without severe toxic effects compared with photon therapy and PBT. However, in this multi-institutional study protocol, patients with a history of chemotherapy before CIRT were excluded, whereas in our study, approximately 70% of patients had a prior history of chemotherapy before PBT. As a result, many patients in our cohort were possibly already refractory to treatment, which might have negatively affected our outcomes. However, there are no guidelines or recommendations for patients with LRRC on whether PBT or CIRT is better, and a randomized study is warranted.

Regarding toxicity, grade 4 late gastrointestinal toxic effects were recorded in 3 patients, in 2 of whom reirradiation was associated with further local recurrence after initial PBT. Therefore, reirradiation for further local recurrence after initial treatment with PBT should not be administered easily owing to the high incidence of gastrointestinal toxic effects. Additionally, clinicians should pay more attention to the radiation dose to the small or large bowel when the patient has a history of bevacizumab use before PBT.

^18^F-FDG-PET/CT is a useful imaging modality that reflects the tumor metabolic activity during cancer treatment. In our study, patients with an SUV_max_ of ^18^F-FDG-PET/CT before PBT that was greater than 10 showed significantly worse OS, PFS, and LC. Uemura et al reported the efficacy of ^18^F-FDG-PET/CT in assessing tumor response to preoperative CRT for LRRC.^20^ They performed ^18^F-FDG-PET/CT before and 3 weeks after CRT (50 Gy/25 fractions photon therapy with irinotecan plus tegafur and uracil) and assessed the SUV_max_ of the pre-CRT scans and post-CRT scans. The mean SUV_max_ after the CRT scan was significantly lower in responders than in nonresponders (*P* = .0038) and was an independent predictor of local recurrence-free survival (*P* = .0383) and OS (*P* = .0195). Moreover, the pathologic response did not correlate with the response as evaluated by CT (*P* > .9999) or MRI (*P* > .9999). In our study, although we could not perform ^18^F-FDG-PET/CT after PBT for all patients (^18^F-FDG-PET/CT was performed in 16 patients, MRI in 4 patients, and CT in 3 patients), the patients who achieved CR or CMR after PBT had significantly better LC than those with non-CR or non-CMR. We believe that the objective assessment of tumor response using ^18^F-FDG-PET/CT is easier to perform than that using CT or MRI. Seventy percent of patients received ^18^F-FDG-PET/CT as initial imaging after PBT to assess the tumor response, which might have enabled us to judge the treatment response correctly. Therefore, ^18^F-FDG-PET/CT before and after PBT may be useful for assessing tumor response and predicting outcomes.

This study had some limitations. First, the number of patients was very small, and the study design was retrospective. Second, the PBT dose and fractionation regimen differed according to the physician in charge. In UVA, although the total radiation dose had no significant correlation with outcomes, variable dose and fractionation regimens would complicate the interpretation of the results. However, to our knowledge, this study is the largest study of patients with LRRC treated with PBT, and these results step forward the treatment for LRRC.

## Conclusion

This study's results showed that PBT may have a potential to be a good treatment option for LRRC. ^18^F-FDG-PET/CT before and after PBT may be useful for assessing tumor response and predicting outcomes.

## Appendix Supplementary materials

Image, application 1

Image, application 2

Image, application 3

## Footnotes

Supplementary material associated with this article can be found in the online version at doi:10.1016/j.adro.2023.101192.
