# Supplementary figures and images for: Outcomes and Prognostic Factors for Locally Recurrent Rectal Cancer Treated With Proton Beam Therapy
Source: Adv Radiat Oncol. 2023 Feb 6;8(3):101192. doi: 10.1016/j.adro.2023.101192 (PMC9991532; doi:10.1016/j.adro.2023.101192)

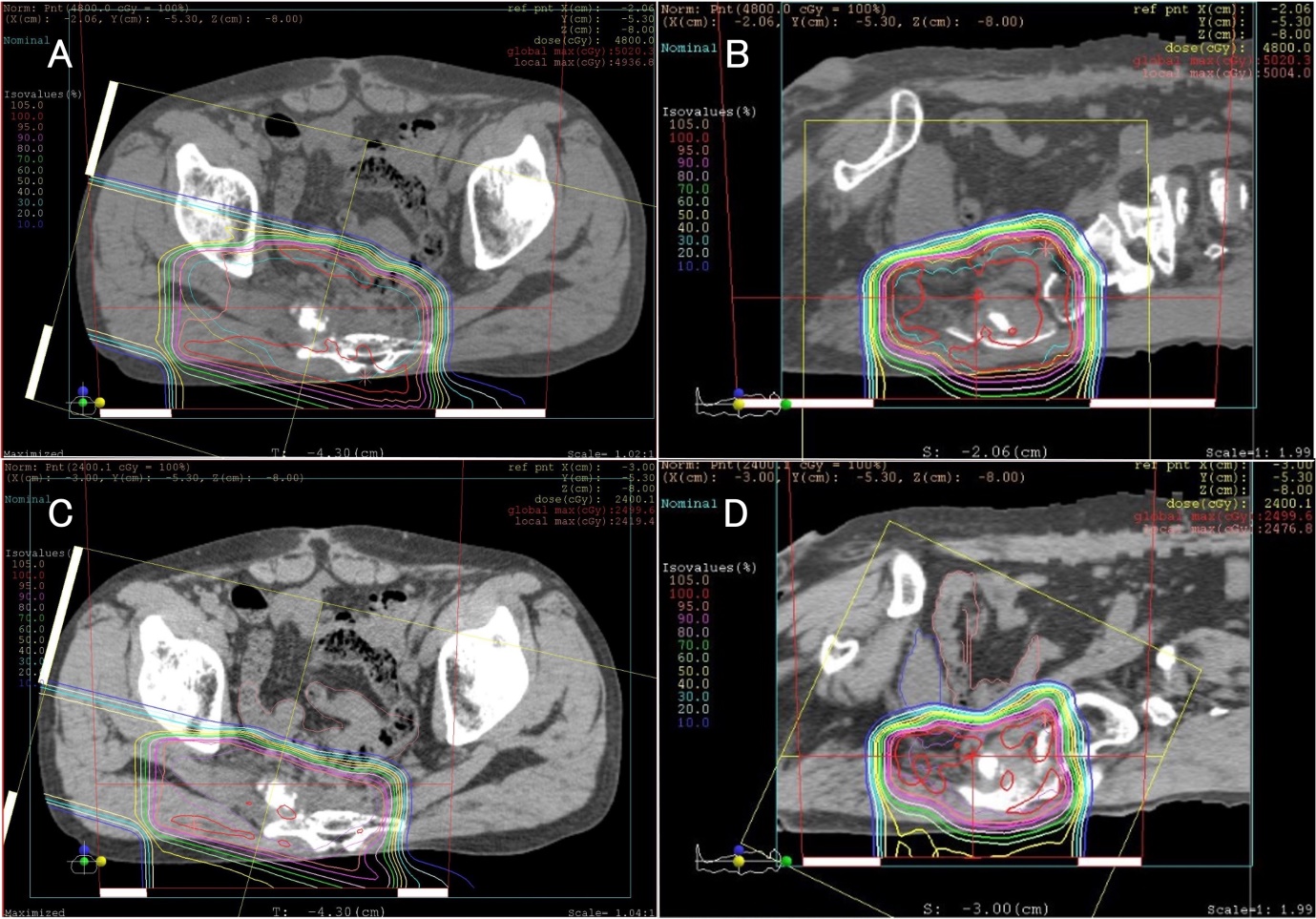

Supplement: Supplementary file 1 [file mmc1.docx]

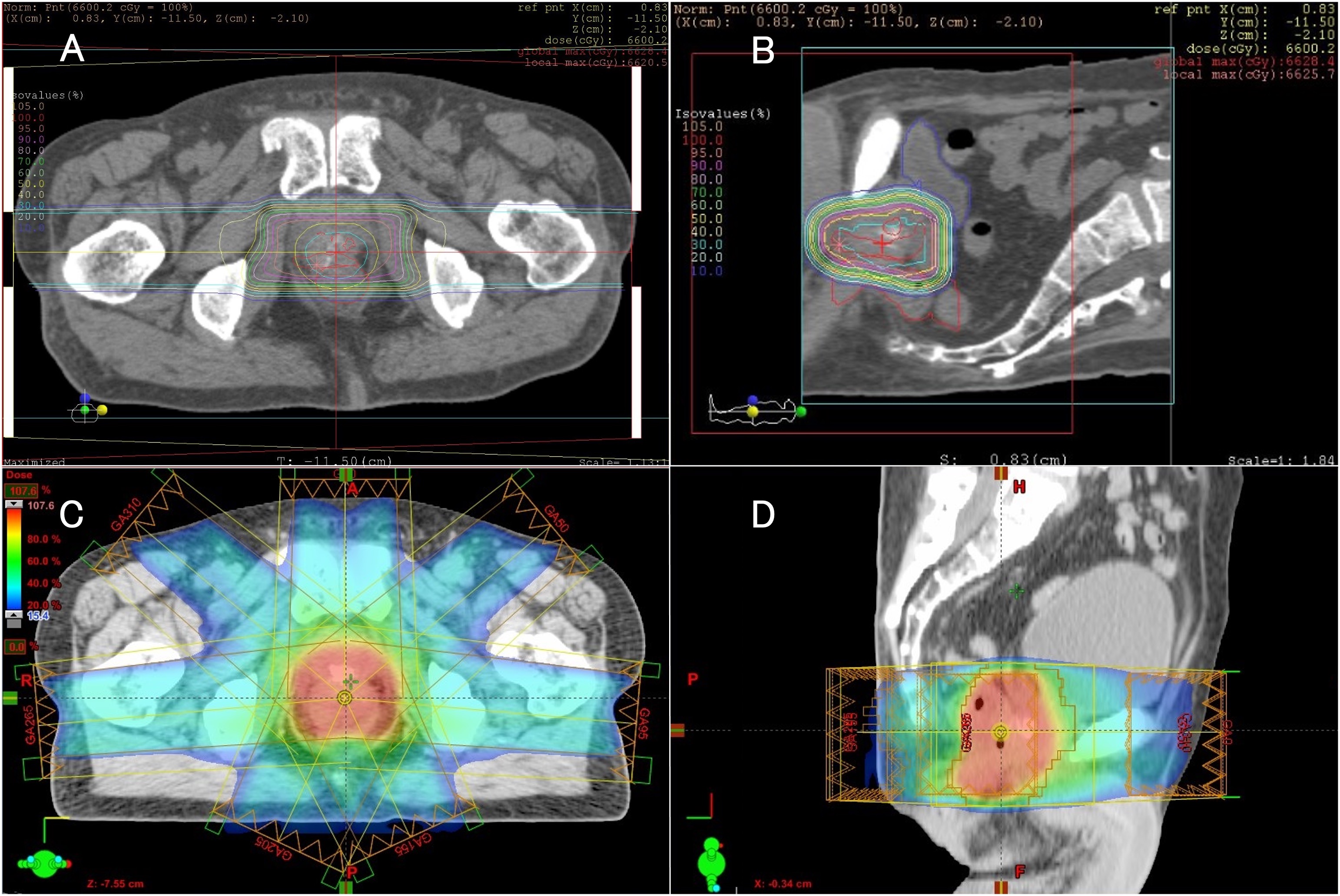

Supplement: Supplementary file 2 [file mmc2.docx]

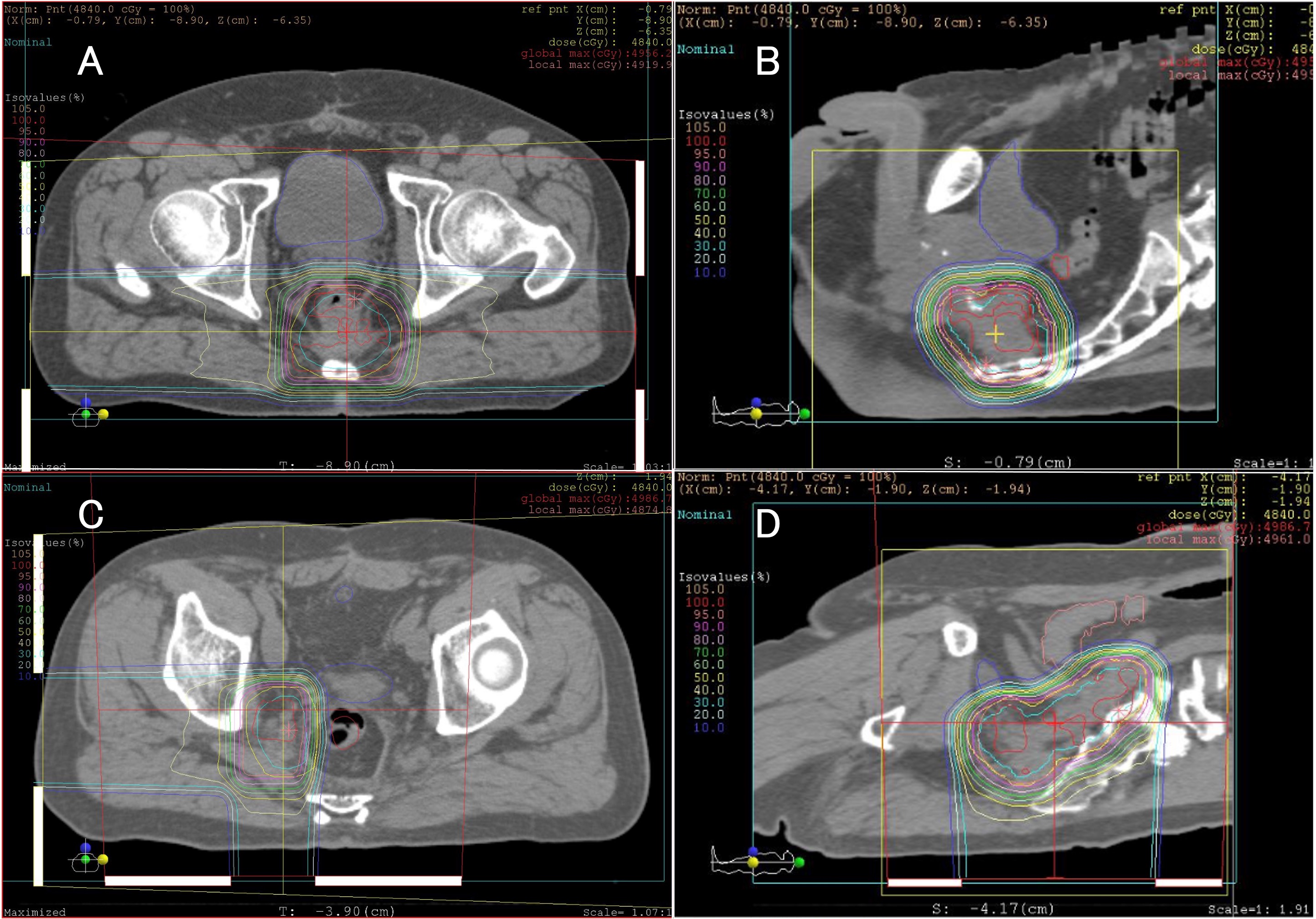

Supplement: Supplementary file 3 [file mmc3.docx]
